# Fermi-Level Tuning of G-Doped Layers

**DOI:** 10.3390/nano11020505

**Published:** 2021-02-17

**Authors:** Avto Tavkhelidze, Amiran Bibilashvili, Larissa Jangidze, Nima E. Gorji

**Affiliations:** 1 Center of Nanotechnology for Renewable Energy, Ilia State University, Cholokashvili Ave. 3-5, Tbilisi, GA 0162, USA; larisajangidze@gmail.com; 2 Institute of Micro and Nano Electronics, Chavchavadze Ave. 13, Tbilisi, GA 0179, USA; amiran.bibilashvili@tsu.ge; 3 School of Physical Sciences, Dublin City University, Dublin 9, Ireland; nima.gorji@dcu.ie

**Keywords:** nanostructuring, semiconductor, doping

## Abstract

Recently, geometry-induced quantum effects were observed in periodic nanostructures. Nanograting (NG) geometry significantly affects the electronic, magnetic, and optical properties of semiconductor layers. Silicon NG layers exhibit geometry-induced doping. In this study, G-doped junctions were fabricated and characterized and the Fermi-level tuning of the G-doped layers by changing the NG depth was investigated. Samples with various indent depths were fabricated using laser interference lithography and a consecutive series of reactive ion etching. Four adjacent areas with NG depths of 10, 20, 30, and 40 nm were prepared on the same chip. A Kelvin probe was used to map the work function and determine the Fermi level of the samples. The G-doping-induced Fermi-level increase was recorded for eight sample sets cut separately from p-, n-, p^+^-, and n^+^-type silicon substrates. The maximum increase in the Fermi level was observed at a10 nm depth, and this decreased with increasing indent depth in the p- and n-type substrates. Particularly, this reduction was more pronounced in the p-type substrates. However, the Fermi-level increase in the n^+^- and p^+^-type substrates was negligible. The obtained results are explained using the G-doping theory and G-doped layer formation mechanism introduced in previous works.

## 1. Introduction

**Figure 1:**
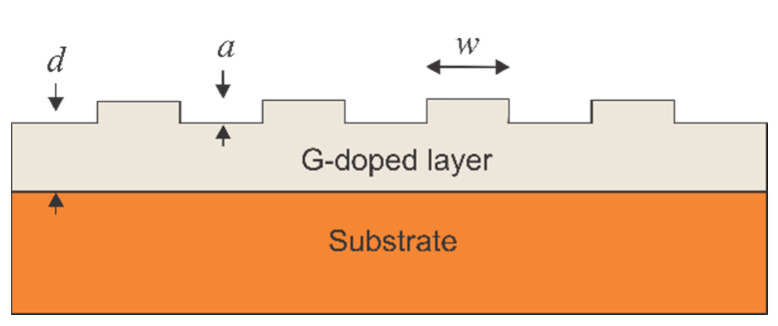
Nanograting layer on a substrate surface and the induced G-doped layer.

The latest developments in nanotechnology have allowed the fabrication of low-dimensional periodic nanostructures [1,2,3], including nanogratings (NGs). Imposed periodic nanostructures such as NG layers are known to significantly affect the electronic [4,5], thermoelectric [6,7], optical [8,9], electron emission [10,11], and magnetic [12,13] properties of semiconductors when the NG depth becomes comparable to the de Broglie wavelength. This can be attributed to the special boundary conditions enforced by the NG on the wave function. This is because they forbid some quantum states [14], thus reducing their density. Figure 1 shows an NG layer fabricated on a semiconductor wafer surface.

In the proximity of the NG within a layer of thickness *d*, the density of the quantum states (DOS) reduces. The boundary conditions enforced by the NG layer influence only the wave functions of electrons that are in close proximity to the surface. In contrast, electrons that are far from the surface decohere. This could be attributed to the loss of coherence of these electrons during scattering events, thus making the grating boundary conditions negligible. 

**Figure 2:**
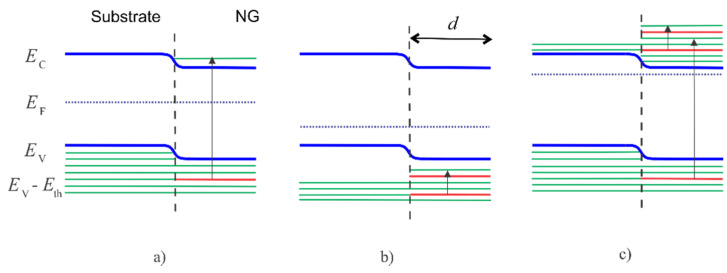
Basic energy-band diagrams of an NG layer fabricated on the surface of (**a**) an i-type semiconductor, (**b**) a p-type semiconductor, and (**c**) an n-type semiconductor. The green lines indicate the levels occupied by electrons and the red lines indicate the levels forbidden by the NG. For simplicity, the band gap is scaled down and the energy levels are shown to be equidistant.

Figure 2 shows the effect of the NG layer on the energy levels of semiconductors. Figure 2a shows a basic energy-band diagram of an i-type semiconductor substrate with an NG layer fabricated on its surface.

As shown in the image, some levels in the valence band are forbidden, thus causing the rejected electrons to occupy higher energy levels in the conduction band. Consequently, the concentration of electrons in the conduction band increases, which is referred to as geometry-induced doping [3]. Figure 2b shows a diagram of a p-type semiconductor substrate. As shown in the image, rejected electrons move to the top of the valence band with empty energy levels. Figure 2c shows an energy-band diagram of an n-type semiconductor substrate. As shown in the image, electrons are rejected from both the valence and conduction bands and move to the bottom of the conduction band with empty energy levels. 

Considering the increase in the Fermi energy, geometry-induced doping (G-doping) is equal to donor doping. For example, in i-type substrates, G-doping forms i-n junctions. However, in p-type substrates it can form incompletely compensated p-p^−^, entirely compensated p-i, or even p-n junctions reliant on the G-doping level. In contrast, in n-type substrates n-n^+^ junctions are formed. 

Several studies have investigated nanostructure-induced n-type doping, such as in disordered structures obtained by the wet etching of p-Si [15]; in almost periodic nanostructures fabricated by the laser radiation of Si, Ge, and SiGe crystals [16]; and in mesoporous p-Si where holes disappear [17,18]. Porous Si/CdS heterojunctions were found to increase in terms of solar cell efficiency [19]. The results of these studies can be explained using the G-doping mechanism. Surface superconductivity changes in Nb sheets induced by nanograting were observed in [20]. Laser irradiation was found to induce nanograting structure on the Au surface [21]. Geometry influence on nanostructure properties was analyzed in [22]. 

Various studies have experimentally investigated thin Si NG layers, and their resistivity and Hall voltage temperature dependences have been confirmed to be metal type [23], indicating a considerable increase in the Fermi level. In addition, the dielectric function of Si NG layers determined by ellipsometry revealed the metallization of the layer [9,23]. Moreover, strong photoluminescence (PL) has been observed in Si NG layers [9] regardless of the indirect band gap, indicating electron space localization, which is consistent with simulations [14] based on the G-doping theory [4]. NG layers fabricated from bulk Si substrates form a G-doped p-p(v) junction [24]. Moreover, experimental analysis results have revealed that the G-doping level of NG layers can be adjusted electrically [25]. 

In this study, we investigated the dependence of the G-doping level on the NG indent depth for p-, n-, p^+^-, and n^+^-type substrates. This is in contrast to previous experiments, where the G-doping-induced built-in potential was calculated from the p-p (V) junction current-voltage (I–V) characteristics [24]. In this study, the built-in potential (work function (WF) difference) was measured directly using a homemade Kelvin probe (on the base of microscope MI-4) and, additionally, n-, p^+^-, n^+^-doped material was investigated. 

## 2. Materials and Methods 

**Figure 3:**
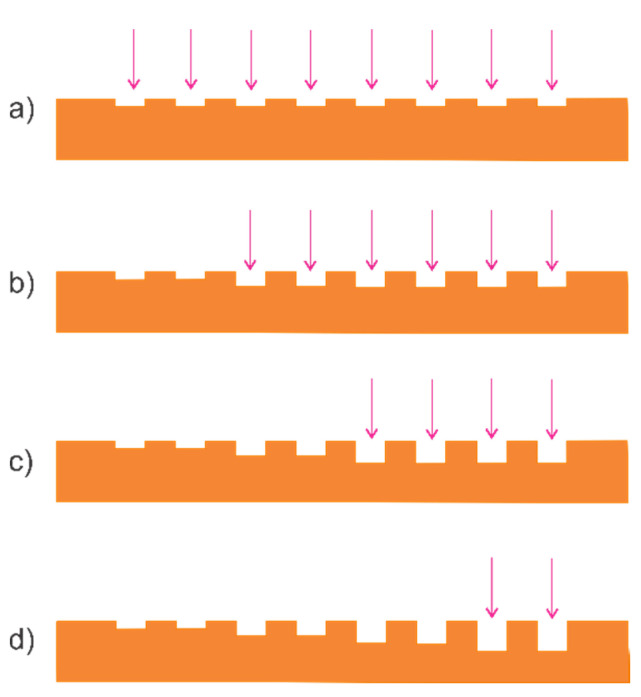
Consecutive reactive ion etching for the NG layer preparation. First RIE etching (**a**) for indent depth of 10 nm, second RIE etching (**b**) for indent depth of 20 nm, third RIE etching (**c**) for indent depth of 30 nm, fourth RIE etching (**d**) for indent depth of 40 nm.

Four Si substrates of different types and doping levels were used in this study: the p-type 1–10 Ω × cm, n-type 1.7–2 Ω × cm, p^+^-type 0.044 Ω × cm, and n^+^-type 0.013 Ω × cm. First, the samples were cut into chips with dimensions of 10 mm × 10 mm. The samples were divided into four groups, each containing eight chips of the p-, n-, p^+^-, and n^+^-type substrates (total of 32 chips). Subsequently, an NG layer with a pitch of 300 nm was fabricated on the chips over an area of 3 mm × 3 mm (at the center of the chip) by laser interference lithography [5], followed by Reactive Ion Etching (RIE). A coherent laser with a wavelength of 375 nm and a Lloyd interferometer were used for the interference lithography. The reactive ion etching of Si was performed using CF_4_, as described in [5,13]. The reactive ion etching time was adjusted to 35 s to achieve an indent depth of 10 nm (Figure 3a). 

Next, part of the NG area was covered with a metallic mask and the reactive ion etching was repeated to obtain an indent depth of 20 nm (Figure 3b). This process was repeated to obtain indent depths of 30 and 40 nm (Figure 3c,d). The indent depth was monitored by scanning a plain area (adjacent to the NG layer) using a profile meter. The cross-section of the NG layer was sinusoidal (see Figure 1 of [13]), which is characteristic of NG layers prepared by laser interference lithography. Finally, a metal was deposited on the back side of the chip to achieve ohmic contact: Al was deposited on the p-type and p^+^-type substrates by thermal evaporation and Ti/Ag was deposited on the n-type and n^+^-type silicon substrates by magnetron sputtering, as described in [24]. After exposing the sample to the air, the native oxide layer was removed (in HF 1:20 solution, for 5 s) and a perchlorovinyl chemical-resistant lacquer was attached to the sample. The chemical-resistant layer was removed directly before WF measurement.

**Figure 4:**
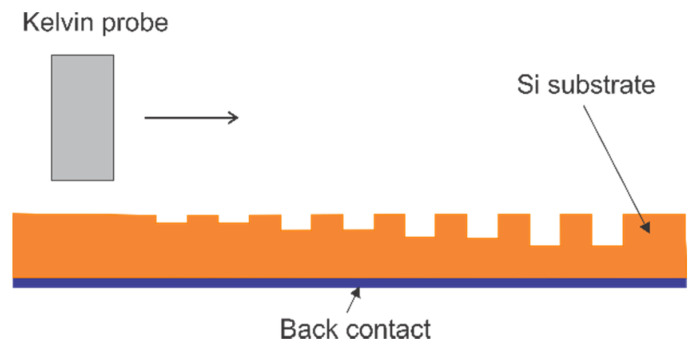
Work function measurement setup.

The WF of the substrates was measured using a Kelvin probe [26]. Briefly, the samples were scanned using a vibrating tungsten needle with a diameter of 500 µm, as shown in Figure 4. The WFs of the plain area and the 10, 20, 30, and 40 nm indent areas were recorded. The WF of the plain area, $\phi_{pn}$, was subtracted from the WF of the NG areas, $\phi_{ng}$, to obtain the corresponding WF difference and characterize the influence of the NG layer. The $\phi_{pn}$ was measured on the corresponding plain areas adjacent to the nanograting. 

## 3. Results and Discussion

**Figure 5:**
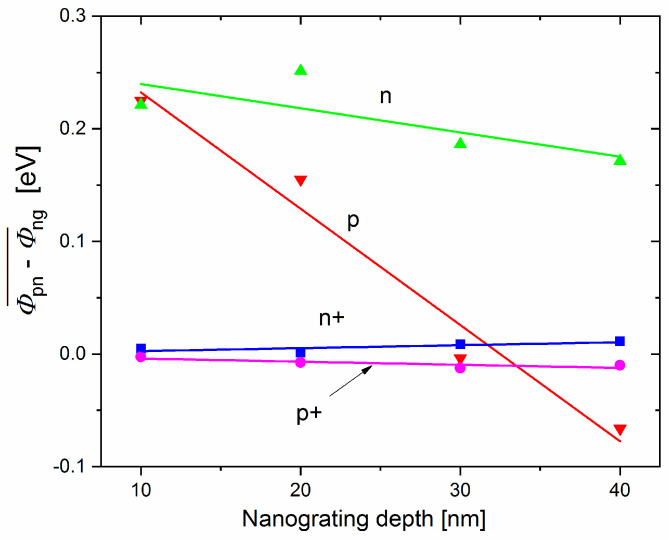
Dependence of the WF difference on the indent depth for the p- (red), n- (green), n^+^- (blue) and p^+^- (violet) type samples. Lines of corresponding colors are linear fits.

The differences in the obtained WF were averaged within the p, n, p^+^, and n^+^ sample groups. The WF difference, $\phi_{pn}-\phi_{ng}$, of a group containing eight samples was averaged to obtain $\bar{\phi_{pn}-\phi_{ng}}$. The standard deviation (SD) within the same group was also calculated using the formula $SD=\sqrt{\sum {\left(\phi_{pn}-\phi_{ng}\right)}^{2}/N}$, where *N* is the number of samples within a group [27]. The dependence of $\bar{\phi_{pn}-\phi_{ng}}$ on the indent depth of the p-, n-, p^+^-, and n^+^-type samples is plotted in Figure 5. The linear fit of the four averaged values (10, 20, 30, and 40 nm) is included for clarity.

As shown in Figure 5, with an increase in the indent depth of the p- and n-type substrates, the WF difference (Fermi-level difference) decreases. In addition, the highest Fermi energy increase was observed at an indent depth of 10 nm for both the p-type and n-type substrates and the WF difference was approximately 0.25 eV. The 0.25 eV rise in the Fermi energy of the p-type samples corresponded to the reduction in the hole concentration from ≈5 × 10^15^ cm^−3^ to ≈1 × 10^12^ cm^−3^. In contrast, the approximately 0.25 eV increase in the Fermi energy of the n-type samples corresponded to an increase in electron concentration from ≈3 × 10^15^ cm^−3^ to ≈1 × 10^18^ cm^−3^. These results indicate that the G-doping level in both samples was sufficiently high to change the substrate type from p to p+ or from n to n^+^. In addition, the G-doping level strongly depends on the indent depth.

As shown in Figure 5, the Fermi-level difference in the p^+^- and n^+^-type substrates at all indent depths is very low. This could be attributed to the high concentration of charge carriers in the substrate (degenerate semiconductors). Consequently, the G-doping rejected electron concentration was not sufficiently high to significantly change the carrier concentration and Fermi level.

Table 1 shows the substrate resistivity and corresponding carrier concentrations of different types of Si substrates obtained from [28].

The p^+^- and n^+^-type substrate carrier concentrations were two to three orders higher than those of the p- and n-types, and thus could not be significantly affected by the concentration of rejected electrons. In addition, the slight increase in the Fermi-level difference of the n^+^ substrates with increasing indent depth falls within the SD. The SD values for each indent depth, as well as the substrate resistivity and the corresponding carrier concentrations, are given in Table 1.

The mapping of steps of the plain area adjacent to the nanograting shows that the WF difference between plain areas of 0, 10, 20, 30, and 40 nm depth was less than 0.1%. This shows that RIE did not introduce a substantial number of surface states. This is consistent with the results obtained in the study of RIE contamination in Si [29]. Our result is easier to explain if we take into account that the etching time was much shorter in our experiments. Alternatively, the shallow surface defects introduced by RIE were detached in the process of native oxide removal.

The lack of influence of the NG layer on the Fermi level of the p^+^- and n^+^-type substrates could also be attributed to the fact that the ionized impurity concentration in the n^+^- and p^+^-type substrates was sufficiently high to suppress quantum interference by decohering charge carriers. The suppression of the quantum interference by impurities has been previously reported in metal NG films [30].

As shown in Table 1, the standard deviation of the n- and p-type (especially for p) substrates was higher than those of the n^+^- and p^+^-type substrate samples. This could be attributed to the resistivity variation along the substrate from which the samples were cut. In addition, the p-type substrate exhibited the highest resistivity variation (one order of magnitude), as given in the substrate specifications.

The obtained results are consistent with the G-doping mechanism. For simplicity, we analyzed an i-type semiconductor (Figure 1) within the parabolic band approximation, after which the results were extrapolated to both the p-type and n-type wafers. The number of rejected electrons is given by the formula [3]:

(1) $$n_{r}^{\left(i\right)}=\left(1-G^{-1}\right)\int_{E_{V}-E_{th}}^{E_{V}}dE\rho_{0}\left(E\right)$$

where *G* is the geometry factor, $\rho_{0}\left(E\right)$ is the DOS of the substrate, and $E_{th}$ is the threshold energy that characterizes the maximum energy at which a reduction in the DOS occurs [25]. As shown in Equation (1), $n_{r}$ depends on the $E_{th}$ and *G*. First, the $E_{th}$ dependence was considered. The integral in Equation (1) equals the number of quantum states in the energy region, $E_{V}-E_{th}<E<E_{V}$. With a decrease in the area of the energy region, the number of rejected electrons decreased. In addition, with an increase in the indent depth, the width of this region reduced. This could be attributed to the NG effect on the DOS. This effect is significant only if the indent depth is close to the de Broglie wavelength. Therefore, electrons with short de Broglie wavelengths (high values of wave number *k*) were not influenced. Consequently, we have a threshold energy value, $E_{th}=(\hbar^{2}/2m)k_{th}^{2}$, at which the DOS was still influenced by the NG. With an increase in the indent depth, the integral in Equation (1), which is proportional to ${\left(E_{th}\right)}^{3/2}$ [3], decreased rapidly, thus decreasing $n_{r}$ and the Fermi energy difference. Consequently, the WF difference, $\phi_{pn}$ − $\phi_{ng}$, was reduced. The above analysis can be easily extrapolated to the p-type substrate (Figure 1). The only difference is that in the p-type substrate, rejected electrons fill the empty quantum states at the top of the valence band instead of transferring to the conduction band. In contrast, the extrapolation to the n-type substrates is a bit more complex. In the n-type substrates, there was one more electron rejecting the energy region at the conduction band bottom. Consequently, the number of rejected electrons increases and becomes:

(2) $$n_{r}^{\left(n\right)}\approx \left(1-G^{-1}\right)\left[\int_{E_{V}-E_{th}}^{E_{V}}dE\rho_{0}\left(E\right)+\int_{E_{C}}^{E_{C}+E_{th}}dE\rho_{0}\left(E\right)\right]$$

Here, we presumed that $E_{th}$ and $\rho_{0}\left(E\right)$ have similar values in the conduction band, which is a very rough approximation as the zone structure is diverse. Nevertheless, Equation (2) is sufficient for the qualitative analysis. Equation (2) indicates that the rejected electron number in the n-type substrate should be higher. Experiments revealed that this was true for the 20, 30, and 40 nm indent depths, but not for the 10 nm indent depth, which shows nearly equal values of $\phi_{pn}$ − $\phi_{ng}$ for the p- and n-type substrates. This divergence could be attributed to the logarithmic dependence of the Fermi level on the carrier concentration. However, with increasing Fermi levels, its dependence on $n_{r}$ becomes less pronounced.

As shown in Equation (2), the number of rejected electrons depends on *G*. According to DOS numerical analysis [31], *G* increases with increasing indent depth in the ad<0.4 range. Using the G-doping value of *d* = 240 nm obtained in [24], we observed that we are well within this range for all indent depths. Consequently, with increasing indent depth, *G* increased, thus increasing its coefficient $\left(1-G^{-1}\right)$. This increase partly compensates for the reduction in the integrals in Equation (2). A detailed quantitative analysis of the rejected electron number can be conducted using the data obtained in [24,31] and the band structure of Si. However, this is beyond the scope of this work.

In summary, the experimental results were consistent with the G-doping theory and revealed that the G-doping level in the Si substrates decreased with increasing indent depth in the range from 10–40 nm. Furthermore, the G-doping level was relatively high in both the n- and p-type substrates and can be easily varied over a wide range by changing the indent depth. In addition, the G-doping level was higher in the n-type substrate. However, the G-doping was not pronounced in the p^+^ and n^+^ substrates. The built-in potential value (for p-type at indent depth of 30 nm) is consistent with the values obtained from the I–V curves in reference [24] and calculated in reference [25]. Theoretically, the built-in potential should increase with decreasing nanograting pitch. We plan to perform the fabrication and characterization of lower-pitch nanogratings and other periodic nanostructures.

## 4. Conclusions

In conclusion, in this study the G-doping level dependence on the NG depth was investigated in p-, n-, p^+^- and n^+^-type Si substrates. The G-doping was induced by NG with a pitch of 300 nm and depths of 10, 20, 30, and 40 nm. The NG layer was fabricated using laser interference lithography and a consecutive series of reactive ion etching. The Fermi energy difference between the plain and NG surfaces was measured using a Kelvin probe. The measurements revealed that the G-doping level reduced with increasing indent depth in the range from 10 to 40 nm for both the p- and n-type substrate samples. However, the indent depth dependence was more pronounced in the p-type substrate samples compared to the n-type substrate specimens. The maximum increase in the Fermi level was ≈0.25 eV for both the p and n-type substrate specimens. This corresponded to a NG-induced change from p to p^−^ or from n to n^+^. However, G-doping was not observed in the p^+^- and n^+^-type substrate samples. This could be attributed to the low concentration of rejected electrons compared to the substrate carrier concentration. The results of this study indicate that NG provides alternative doping methods that do not require ionized impurities, that can be controlled by changing the indent depth, and that can work in a varied range of doping levels. These results will increase the applications of G-doping in electronic and optoelectronic devices. 

## Figures and Tables

**Table 1:** Standard deviation (SD) dependence on the substrate type, doping level, and indent depth. Substrate resistivity and carrier concentrations are included for clarity.

Substrate Type	p	n	p^+^	n^+^
Substrate resistivity [Ω × cm]	1–10	1.7–2	0.044	0.013
Substrate carrier concentration [cm^−3^]	1.5 × 10^16^ –1.5 × 10^15^	3 × 10^15^	1 × 10^18^	3 × 10^18^
SD for *a* = 10 nm [eV]	1.6 × 10^−1^	3.1 × 10^−2^	2.7 × 10^−2^	2.1 × 10^−2^
SD for *a* = 20 nm [eV]	1.2 × 10^−1^	3.5 × 10^−2^	1.7 × 10^−2^	2.7 × 10^−2^
SD for *a* = 30 nm [eV]	5.8 × 10^−2^	3.5 × 10^−2^	2.5 × 10^−2^	3.0 × 10^−2^
SD for *a* = 30 nm [eV]	5.8 × 10^−2^	3.5 × 10^−2^	2.5 × 10^−2^	3.0 × 10^−2^

## Data Availability

Data sharing not applicable.
